# Comprehensive analysis of DNA methylation and gene expression in orally tolerized T cells

**DOI:** 10.1371/journal.pone.0229042

**Published:** 2020-02-25

**Authors:** Ayano Toyoda, Toshinori Kozaki, Kazuo Ishii, Momoka Taniishi, Makoto Hattori, Hiroshi Matsuda, Tadashi Yoshida

**Affiliations:** 1 Department of Applied Biological Science, Tokyo University of Agriculture and Technology, Fuchu, Tokyo, Japan; 2 Biostatistics Center, Kurume University, Kurume, Fukuoka, Japan; 3 Division of Animal Life Science, Tokyo University of Agriculture and Technology, Fuchu, Tokyo, Japan; Wayne State University, UNITED STATES

## Abstract

T cell anergy is known to be a crucial mechanism for various types of immune tolerance, including oral tolerance. The expression of several anergy-specific genes was reportedly up-regulated in anergic T cells, and played important roles in the cells. However, how the genes were up-regulated has not been understood. In this study, we comprehensively analyzed the altered gene expression and DNA methylation status in T cells tolerized by oral antigen *in vivo*. Our results showed that many genes were significantly up-regulated in the orally tolerized T cells, and most of the genes found in this study have not been reported previously as anergy related genes; for example, ribosomal protein L41 (FC = 3.54E06, *p* = 3.70E-09: Fisher's exact test; the same applies hereinafter) and CD52 (FC = 2.18E05, *p* = 3.44E-06). Furthermore, we showed that the DNA methylation statuses of many genes; for example, enoyl-coenzyme A delta isomerase 3 (FC = 3.62E-01, *p* = 3.01E-02) and leucine zipper protein 1 (FC = 4.80E-01, *p* = 3.25E-02), including the ones distinctly expressed in tolerized T cells; for example, latexin (FC = 3.85E03, *p* = 4.06E-02 for expression; FC = 7.75E-01, *p* = 4.13E-01 for DNA methylation) and small nuclear ribonucleoprotein polypeptide F (FC = 3.12E04, *p* = 4.46E-04 for expression; FC = 8.56E-01, *p* = 5.15E-01 for DNA methylation), changed during tolerization, suggesting that the distinct expression of some genes was epigenetically regulated in the tolerized T cells. This study would contribute to providing a novel clue to the fine understanding of the mechanism for T cell anergy and oral tolerance.

## Introduction

Oral administration of food antigens is known to induce oral tolerance, and T cell anergy is reported as a major mechanism of oral tolerance as well as other various types of immunological tolerance [1–3]. Anergic T cells do not respond to the relevant antigen stimulation, while surviving for a long period of time. Although many studies have previously reported that the expression of several anergy-specific genes was up-regulated in anergic T cells [4–7], the mechanism for the regulation of their expression remains unknown.

As described above, the increased expression of anergy-specific genes is maintained over a long term [4–7]. Therefore, it has been suggested that some epigenetic regulations may be involved in the regulation of anergy-specific genes [8]; however, there is little evidence to support this proposal. However, given that there are numerous genes showing altered expression levels in anergic T cells, it is unlikely that all the genes are independently and epigenetically regulated. Therefore, we are considering that only a few anergy-specific genes are epigenetically regulated and control the expression of other anergy-specific gene expressions. Indeed, in the case of other T cell subsets, a certain critical gene acts as a master regulator for each respective subset; for example, T-bet, GATA-3, RORγt and Foxp3 for Th1, Th2, Th17 and Treg cells [9–11], respectively. It is expected that the induction of T cell anergy is also regulated by a putative master regulator. In addition, some of the former four have been suggested to be epigenetically regulated [12], suggesting that epigenetic regulation is critical to controlling the regulators’ expression.

We had performed a transcriptome analysis and a genome-wide DNA methylation analysis of T cells that were anergized *in vitro* using the next-generation sequencing technique [13]. Consequently, we found that the expressions of many genes were changed by anergy induction; for example, neuritin 1 (FC = 2.82, *p* = 1.08E-03: Fisher's exact test; the same applies hereinafter) and acid-sensing (proton-gated) ion channel 3 (FC = 2.72, *p* = 7.79E-07), and that the DNA methylation status of some of those genes was also changed; for example, neuritin 1 (FC = 3.00E-01). Based on the results of the study, we have not identified any master regulators of anergic T cells yet; however, the observations do indicate that the induction of T cell anergy is regulated by some epigenetic mechanisms.

In the present study, we performed a transcriptome analysis and a genome-wide DNA methylation analysis using T cells tolerized by oral tolerance as well as the previous study using *in vitro* anergized T cells. We considered that the orally tolerized T cell population included anergic T cells to a certain extent. In the current study, we carried out the study in two purposes; first, we aimed to identify candidates for the master regulator of anergic T cells induced by oral tolerance. Second, we aimed to confirm if the candidates would correspond to those obtained from the previous *in vitro* study [13]. Our present results provide several novel evidences about the features of *in vivo* tolerized T cells and some important clues to understand the mechanism for anergy induction.

## Materials and methods

### Mice

Ovalbumin (OVA)-specific TCR-transgenic DO11.10 mice were obtained from the Jackson Laboratory (Bar Harbor, ME, USA). Their offspring were used at 5–7 weeks of age. The T cells of these mice recognize OVA 323–339 restricted to I-A^d^. The TCR-transgenic mice were bred in the animal facility of our university and were maintained on irradiated food and autoclaved distilled water. All mice were maintained and used in accordance with the guidelines for the care and use of experimental animals of Tokyo University of Agriculture and Technology. The procedures of this study were approved by the animal care and use committee in Tokyo University of Agriculture and Technology (26–8, April 1^st^, 2014; 27–3, April 17^th^, 2015; 28–3, April 19^th^, 2016; 30–6, April 6^th^, 2018). We checked the mice almost every day and confirmed no adverse clinical signs through the experimental periods. Splenocytes were prepared from the mice immediately after sacrificing them by cervical dislocation.

### Induction of oral tolerance

Oral tolerance was induced in mice against OVA by feeding them 20% OVA-containing diet (Oriental Yeast Co., Ltd, Tokyo, Japan) for 7 days.

### Restimulation

The splenocytes from the oral tolerance-induced mice were restimulated by incubation (2 × 10^6^ cells/mL) in the presence of OVA (100 μg/mL) for 3 days. OVA was obtained from Sigma (St Louis, MO, USA).

### ELISA

The supernatant was collected from each well 3 days after the restimulation procedure and was assayed by ELISA for its cytokine concentration. To measure IFN-γ and IL-4 concentrations, Maxisorp immunoplates were coated with purified R4-6A2 rat anti-mouse IFN-γ or purified 11B11 rat anti-mouse IL-4 antibody (BD Pharmingen, San Diego, CA, USA). Each sample and the standard were added after washing and blocking. The bound IFN-γ or IL-4 was detected using XMG1.2 biotinylated rat anti-mouse IFN-γ or BVD6-24G2 biotinylated rat anti-mouse IL-4 (BD Pharmingen) before incubating with alkaline phosphatase–streptavidin. The enzyme substrate—*p*-nitrophenol phosphate—was added, and the absorbance was determined at 405 nm.

To evaluate cell proliferation, splenocytes were also collected from each well 3 days after the restimulation procedure, and the cell proliferation level was measured by Cell proliferation ELISA, BrdU kit (Roche Diagnostics, Basel, Switzerland).

### Purification of T cells

T cells were purified from cultured splenocytes (for transcriptome analysis) or fresh splenocytes (for DNA methylation analysis and RT-PCR) by the MACS separation system (Miltenyi Biotec, Bergisch Gladbach, Germany) using CD90.2 micro beads and an LS column.

### Transcriptome analysis

To prepare cDNA library, total RNA was isolated from normal or tolerized T cells 10 h after restimulation using the Tripure isolation reagent (Roche). To avoid DNA contamination, total RNA was treated with DNase (Takara Bio, Kusatsu, Japan). The cDNA libraries were synthesized using mRNA template and random hexamer primers; then, a custom second-strand synthesis buffer (Illumina, San Diego, CA, USA), dNTPs, RNase H, and DNA polymerase I were added to initiate the second-strand synthesis. After a series of terminal repair, a ligation and sequencing adaptor ligation, the double-stranded cDNA libraries were completed through size selection and PCR enrichment. The qualified libraries were fed into an Illumina HiSeq sequencer after pooling according to its effective concentration and the expected data volume.

The quality of the sequencing reads was checked by FastQC (Babraham Bioinformatics, Cambridgeshire, UK), and any low-quality sequences were trimmed by a custom-made script with Perl, v5.10.1. The trimmed sequence reads were subjected to further analyses. The reads for RNA-Seq were mapped onto mouse cDNA reference sequences (Mus_musculus.GRCm38.cdna.abinitio.fa; ftp.ensembl.org) by Bowtie2 (version 2.0.6) [14]. The mapped reads were counted and calculated as FPKM. All statistical analyses were performed by GNU bash (version 4.3.48(1)-release) and R (version 3.4.3). Statistical significance of differential expression was represented by *p*-value with Fisher’s exact test. Biological annotation and analyses, including Gene Ontology (GO) analysis and KEGG pathways analysis, were performed with DAVID Bioinformatics Resources 6.8 software (https://david.ncifcrf.gov/).

### RT-PCR

cDNA was synthesized from total RNA obtained from restimulated normal or tolerized T cells by using a Transcriptor First Strand cDNA Synthesis kit (Roche), and qPCR was then performed (Thermal Cycler Dice Real Time System II, Takara Bio, Kusatsu, Japan) by using KAPA SYBR FAST qPCR kit (KAPA Biosystems, Wilmington, MA, USA). The level of expression of GRAIL was standardized by that of β-actin. The specific primers for each gene are listed as follows: β-actin, 5’-GTGGGCCGCTCTAGGCACCAA-3’ and 5’-CTCTTTGATGTCACGCACGATTTC-3’; GRAIL, 5’-AGAGAGAGGGGCTTCTGGAG-3’ and 5’-CGATGACCATTGTGACTTGG-3’. The statistical analysis was performed by Student’s t test.

### Bisulfite treatment of genomic DNA

To analyze the changes in the DNA methylation status owing to the induction of oral tolerance, genomic DNA was prepared from the normal and tolerized T cells by the phenol–chloroform method. The DNA sample was treated with bisulfite using a MethylEasy Xceed Rapid DNA Bisulphite Modification Kit (Takara Bio). The completion of the bisulfite treatment was confirmed by PCR using Epitaq HS (for bisulphite-treated DNA, Takara Bio) according to the manufacturer’s protocol.

### DNA methylation analysis

The DNA samples that were or were not treated with bisulfite were sequenced by an Illumina GAIIx sequencer using an TruSeq DNA Methylation kit (Illumina) according to the manufacturer’s protocol.

Sequence reading of bisulfite-genome sequencing for DNA methylation analysis was performed with mouse genomic DNA reference sequences (Mus_musculus.GRCm38.dna.toplevel.fa; ftp.ensembl.org) by Bismark (Babraham Bioinformatics, Cambridgeshire, UK) [15]. Annotation and statistical analyses for DNA methylation analysis were performed by using a gff file (Mus_musculus.GRCm38.84.gff3; ftp.ensembl.org) by custom-made scripts with GNU bash (version 4.3.48(1)-release), Perl (v5.22.1), and R (version 3.4.3). Statistical significance of differential methylation level was represented by *p*-value with Fisher’s exact test. Biological annotation and analyses, including Gene Ontology (GO) analysis and KEGG pathway analysis, were performed by DAVID Bioinformatics Resources 6.8 software. The methylation rate of cytosines within 10 kb upstream from the transcription start site (TSS) of each gene was analyzed.

## Results

### Orally tolerized T cells specifically expressed various genes

**Fig 1:**
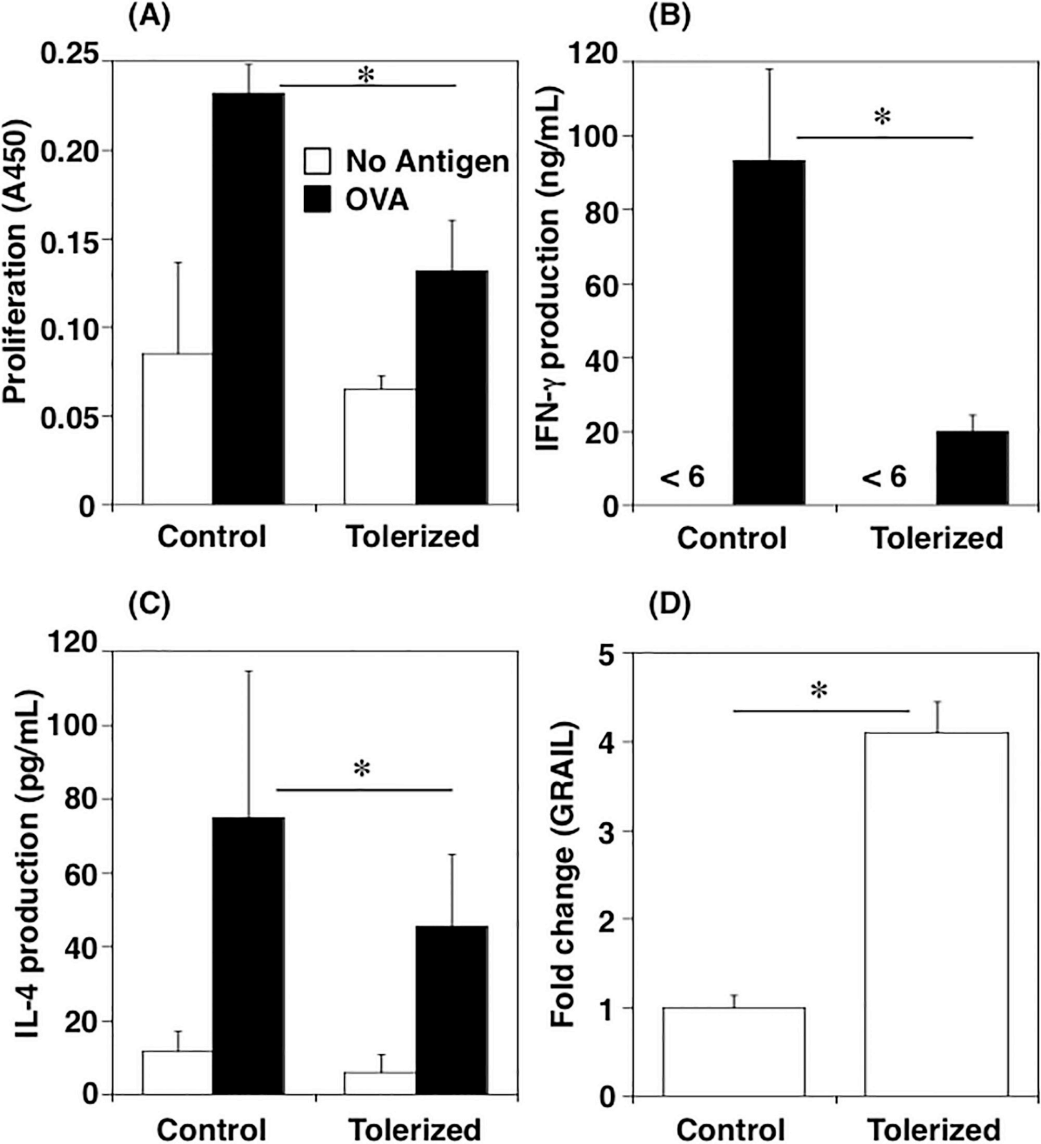
T cell unresponsiveness in orally tolerized mice. Splenocytes from DO11.10 mice that had been fed OVA-containing diet for 7 days were cultured with OVA (100 μg/mL). The proliferation and cytokine production were measured at day 3 of the culture. (A) Proliferation, (B) IFN-γ, (C) IL-4. The expression of GRAIL were analyzed at 18 hours after restimulation. The expression was standardized by β-actin, and the result was shown in (D) as fold change (vs Control). The results were obtained using 3 mice per group. The error bars indicate standard error. * indicates *p*<0.05: Fisher's exact test.

**Fig 2:**
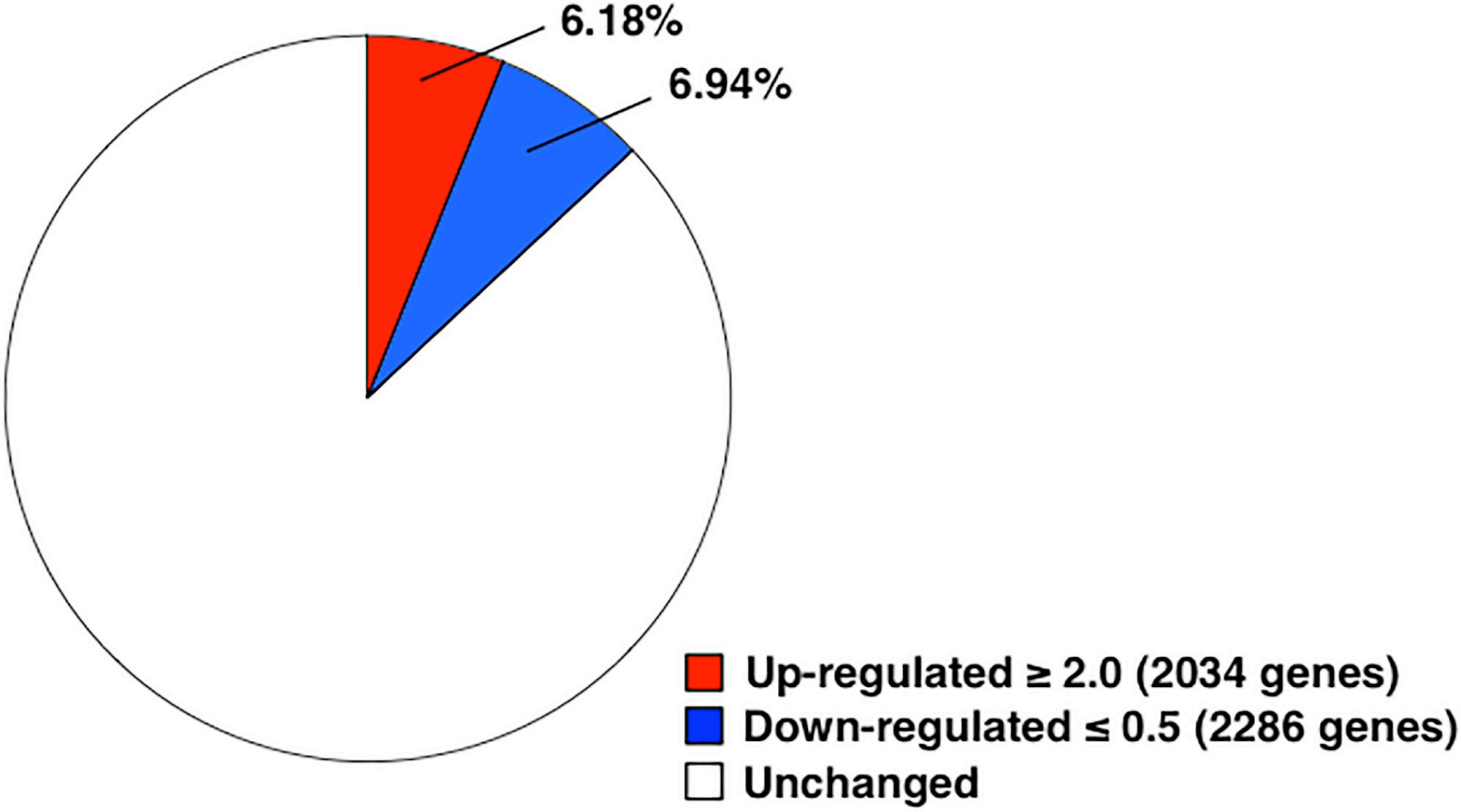
Percentage of genes with up- or down- regulated expression during tolerization. Splenocytes from DO11.10 mice that had been fed OVA-containing diet for 7 days were restimulated with OVA. Total RNA was prepared from the cells 10 h after the restimulation, and underwent transcriptome analysis. Genes with fold change rate ≥2.0 were considered to be up-regulated, while those with fold change rate ≤0.5 were considered to be down-regulated.

To identify the genes specifically expressed in orally tolerized T cells, the cells were subjected to transcriptome analysis. We first confirmed the induction of tolerization in the splenocytes of the orally tolerized mice [1,4]. Our results showed that all four parameters—proliferation (Fig 1A), IFN-γ (Fig 1B) and IL-4 (Fig 1C) production were significantly suppressed, and GRAIL expression (Fig 1D) was significantly up-regulated—were changed in the tolerized T cells. Next, we prepared the mRNA samples and libraries for transcriptome analysis using T cells purified from the antigen-restimulated splenocytes. We confirmed that the purity of the T cells was approximately 90%. We also confirmed that the frequency of OVA-specific T cells in DO11.10 was approximately 65%. We analyzed three samples obtained from different mice for each group and showed the data regarding the number of reads obtained from DNA sequencing for transcriptome analysis (S1 Table). We defined the altered expression of genes caused by tolerization according to the following criteria: increased by 2.0-fold or more, or decreased to 0.5-fold or less. Our results showed that we could detect the expression of 32926 genes, and that the expressions of 13.02% of the genes were changed in orally tolerized T cells. Among them, 2034 genes; S2 Table (6.18% of them) were increased and 2286 genes (6.94%) were decreased (Fig 2). To understand the biological features of orally tolerized T cells, we analyzed the functions of the 2034 genes that showed increased expression in the tolerized T cells using DAVID Bioinformatics Resources 6.8. Consequently, we found 858 genes on the DAVID database and categorized them into 107 clusters. We have shown the top 5 of them in Table 1. Although DAVID does not give us the name of each cluster, the results would show that clusters 1 and 2 included genes involved in regulation of gene expression by remodeling the chromatin structure, cluster 3 included genes involved in host defense against infections, cluster 4 included genes involved in general immune responses, and cluster 5 included genes involved in binding the metal ions. To identify enriched pathways, we performed the KEGG (Kyoto Encyclopedia of Genes and Genomes) analysis using DAVID Bioinformatics Resources 6.8. We confirmed 1360 genes were assigned by the KEGG analysis among genes with up-regulated expression in the tolerized T cells by 2.0-fold or more. The result showed that several pathways including those involved in immune responses, for example ‘Cytokine-cytokine receptor interaction’ (Fold enrichment = 2.16, *p* = 1.14E-4), ‘Chemokine signaling pathway’ (Fold enrichment = 2.05, *p* = 1.79E-3) and ‘TGF-beta signaling pathway’ (Fold enrichment = 2.46, *p* = 8.75E-3), were changed in the tolerized T cells (Table 2). Next, we analyzed the genes up-regulated in the tolerized T cells in higher detail. In Table 3, we show the genes selected among 121 genes with significance by Fisher’s exact test by following 2 criteria: 1) the top 10 for fold change of expression, or 2) detected in all three tolerized samples. In addition to those genes, we also focused on transcription factors. This list includes several interesting genes, for example PAPA-1 (FC = 1.70E04, *p* = 1.60E-03) and Hexim 1 (FC = 4.14E04, *p* = 2.08E-04), which are known to be involved in the cell cycle regulation, and Hspe1 (FC = 1.20E05, *p* = 1.59E-05), Cd52 (FC = 2.18E05, *p* = 3.44E-06), Ier3 (FC = 7.74, *p* = 4.75E-02), and C1qbp (FC = 2.82E04, *p* = 4.97E-04), which work in immune regulation.

**Table 1:** Functional analysis of genes of which expression was increased during tolerization.

**Annotation Cluster 1**	**Enrichment Score: 8.47**		**Gene Counts**	*P* value*
	CC	nucleosome	29	2.80E-16
	BP	nucleosome assembly	21	9.80E-10
	BP	chromatin silencing	12	1.60E-05
	CC	nuclear chromatin	18	6.10E-03
**Annotation Cluster 2**	**Enrichment Score: 3.09**		**Gene Counts**	***P* value**
	BP	nucleosome assembly	21	9.80E-10
	BP	DNA methylation on cytosine	9	1.50E-05
	BP	DNA replication-dependent nucleosome assembly	9	1.90E-05
	BP	positive regulation of gene expression, epigenetic	9	1.90E-05
	CC	nuclear chromosome	11	2.50E-05
	BP	chromatin silencing at rDNA	10	1.60E-05
	BP	protein heterotetramerization	9	2.70E-04
	BP	regulation of gene silencing	5	9.70E-04
	MF	histone binding	12	6.40E-03
	BP	xenophagy	10	1.30E-02
	MF	nucleosomal DNA binding	6	1.70E-02
	CC	nuclear chromosome, telomeric region	11	2.20E-02
	BP	negative regulation of megakaryocyte differentiation	4	2.30E-02
	BP	positive regulation of defense response to virus by host	10	3.50E-02
**Annotation Cluster 3**	**Enrichment Score: 2.75**		**Gene Counts**	***P* value**
	CC	nuclear nucleosome	11	4.30E-06
	BP	innate immune response in mucosa	5	6.40E-03
	BP	antibacterial humoral response	6	6.50E-03
	BP	defense response to Gram-positive bacterium	9	2.00E-02
**Annotation Cluster 4**	**Enrichment Score: 2.68**		**Gene Counts**	***P* value**
	MF	cytokine activity	22	4.90E-05
	MF	chemokine activity	10	5.80E-05
	BP	chemotaxis	15	1.00E-04
	BP	chemokine-mediated signaling pathway	10	1.60E-04
	BP	immune response	24	1.70E-04
	BP	neutrophil chemotaxis	10	8.80E-04
	BP	inflammatory response	26	8.90E-04
	BP	positive regulation of leukocyte chemotaxis	5	1.80E-03
	BP	cell chemotaxis	10	2.10E-03
	BP	positive regulation of inflammatory response	8	7.90E-03
	BP	cellular response to interleukin-1	9	8.80E-03
	MF	CXCR3 chemokine receptor binding	3	1.30E-02
**Annotation Cluster 5**	**Enrichment Score: 2.12**		**Gene Counts**	***P* value**
	BP	response to metal ion	4	8.40E-03
	BP	cellular zinc ion homeostasis	4	2.60E-02

The functions of genes with increased expression in the tolerized T cells were analyzed by DAVID Bioinformatics Resources 6.8. We found 858 genes on the DAVID database and categorized them into 107 clusters. We have shown the top 5 among them in this Table. MF: Molecular Function, BP: Biological Process, CC: Cellular Component.

*Statistical significance of gene enrichment was evaluated by *P* Value with Fisher’s exact test.

**Table 2:** The enriched KEGG pathways for up-regulated genes in the tolerized T cells.

KEGG entry ID	KEGG pathway	Annotated gene number	*P* value*	Fold enrichment
mmu05322	Systemic lupus erythematosus	39	8.97E-16	4.62
mmu05034	Alcoholism	42	4.85E-13	3.63
mmu04060	Cytokine-cytokine receptor interaction	30	1.14E-04	2.16
mmu05217	Basal cell carcinoma	12	1.93E-04	3.87
mmu05202	Transcriptional misregulation in cancer	22	4.43E-04	2.33
mmu05143	African trypanosomiasis	9	6.44E-04	4.48
mmu04062	Chemokine signaling pathway	23	1.79E-03	2.05
mmu04744	Phototransduction	7	2.93E-03	4.70
mmu04916	Melanogenesis	14	4.09E-03	2.47
mmu04550	Signaling pathways regulating pluripotency of stem cells	17	5.30E-03	2.15
mmu05144	Malaria	9	5.38E-03	3.27
mmu04350	TGF-beta signaling pathway	12	8.75E-03	2.46
mmu04390	Hippo signaling pathway	17	1.23E-02	1.96
mmu05200	Pathways in cancer	34	1.75E-02	1.50
mmu05150	Staphylococcus aureus infection	8	2.26E-02	2.79
mmu04610	Complement and coagulation cascades	10	2.86E-02	2.29
mmu00590	Arachidonic acid metabolism	11	3.01E-02	2.16
mmu04913	Ovarian steroidogenesis	8	4.27E-02	2.45
mmu04022	cGMP-PKG signaling pathway	16	4.59E-02	1.71
mmu04614	Renin-angiotensin system	6	4.71E-02	2.99
mmu04924	Renin secretion	9	4.87E-02	2.21
mmu04971	Gastric acid secretion	9	5.22E-02	2.18
mmu04540	Gap junction	10	5.64E-02	2.03
mmu04530	Tight junction	10	7.11E-02	1.94
mmu05205	Proteoglycans in cancer	18	7.29E-02	1.55
mmu04115	p53 signaling pathway	8	8.70E-02	2.08
mmu00920	Sulfur metabolism	3	9.00E-02	5.81

*Statistical significance of gene enrichment was evaluated by *P* Value with Fisher’s exact test.

**Table 3:** Specific genes up-regulated in the tolerized T cells.

Gene name	Fold Change (Anergy/Control)	*P* value	CV	*
ribosomal protein L41 (Rpl41)	3539867.31	3.70E-09	0.127	a
ribosomal protein S23 (Rps23)	356495.14	1.03E-06	1.732	a
CD52 antigen (Cd52)	218176.05	3.44E-06	1.732	a
translational machinery associated 7 (Tma7)	184700.00	5.57E-06	0.869	a
histidine triad nucleotide-binding protein 1 (Hint 1)	161018.44	7.17E-06	0.871	a, c
Gm5735	131068.93	1.17E-05	1.732	a
heat shock protein 1 (chaperonin 10) (Hspe1)	120268.28	1.59E-05	0.916	a
RIKEN cDNA 2010107E04 gene (2010107E04Rik)	103179.28	2.46E-05	1.732	a
histone cluster 1, H2ai (Hist1h2ai)	78820.71	4.75E-05	1.732	a
histocompatibility 2 K region locus 2	78273.46	4.11E-05	1.732	a
immediate early response 3 (Ier3)	7.74	4.75E-02	0.307	b
CDC28 protein kinase regulatory subunit 2 (Cks2)	5616.69	2.28E-02	1.732	c
INO80 complex subunit B (PAPA-1)	16967.08	1.60E-03	1.732	c
RE1-silencing transcription factor (Rest)	4033.39	4.06E-02	1.732	c
YY1-associated factor 2 (Yaf2)	3725.21	4.06E-02	1.732	c
coiled-coil-helix-coiled-coil-helix domain containing 2 (Chchd2)	42988.99	2.08E-04	1.732	c
complement component 1, q subcomponent-binding protein (C1qbp)	28177.70	4.97E-04	1.732	b, c
hexamethylene bis-acetamide inducible 1 (Hexim1)	41354.78	2.08E-04	0.089	b, c
limb-bud and heart (Lbh)	11919.67	4.16E-03	1.732	c
mediator complex subunit 10 (Med10)	14013.46	2.46E-03	1.732	c
mediator complex subunit 9 (Med9)	6170.381	1.59E-02	0.866	c
polymerase (DNA directed), epsilon 3 (p17 subunit) (Pole3)	5059.61	2.28E-02	1.732	c

Each gene with increased expression in the tolerized T cells was selected based on several criteria.

* a: top 10 genes with higher fold change, b: genes expressed in all 3 tolerized samples, c: genes which have functions of transcription regulation

### The methylation status of various genes altered by tolerization

**Fig 3:**
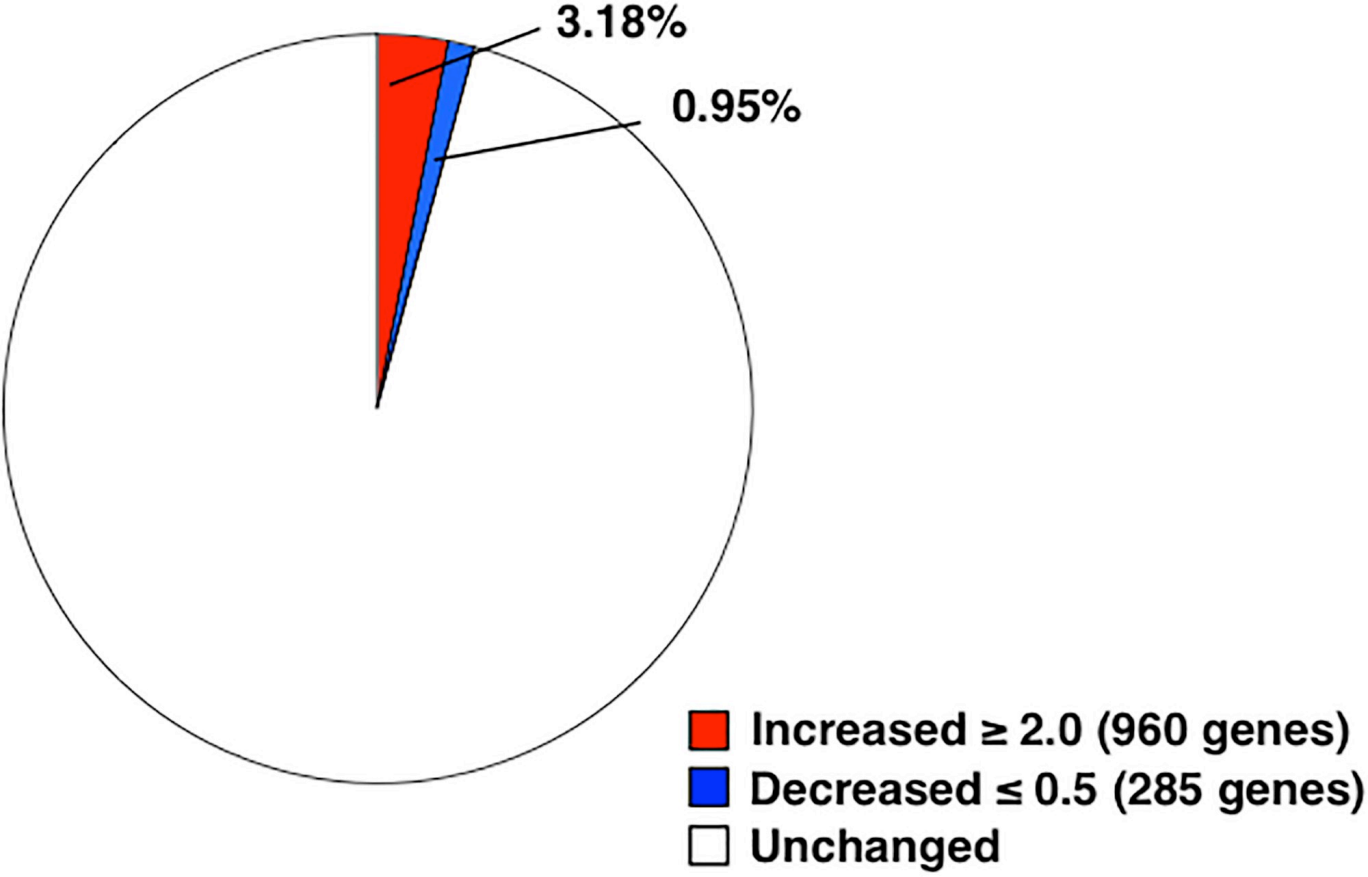
Percentage of genes with up- or down- regulated DNA methylation status during tolerization. DNA was prepared from the splenocytes from DO11.10 mice that had been fed OVA-containing diet for 7 days; it was subjected to DNA methylation analysis after bisulfite treatment. Genes with DNA methylation status increase ≥ 2.0-fold were considered to be up-regulated, while those with the value ≤0.5 were considered to be down-regulated.

Then, we comprehensively analyzed the genome-wide methylation status in both control and orally tolerized T cells. We subjected the DNA samples prepared from the tolerized T cells to DNA sequencing after treating the DNA with bisulfite. We analyzed three samples obtained from different mice for each group and showed the data regarding the number of reads obtained from the sequencing in S3 Table. Tolerization-induced alteration in the methylation status of the genes was defined according to following criteria: increased by 2.0-fold or more, or decreased to 0.5-fold or less. Our results showed that among the identified whole genes (30136 genes), 4.13% (1245 genes) of the genes showed altered DNA methylation status, in orally tolerized T cells; among them, 3.18% of the genes (960 genes) showed increased and 0.95% (285 genes; S4 Table in red characters) showed decreased DNA methylation (Fig 3). Then, we used DAVID to analyze the functions of 285 genes with decreased methylation status in the tolerized T cells. We found 167 genes on the DAVID database and categorized them into 24 clusters. We have shown top 5 of them in Table 4. Clusters 1 and 2 included genes involved in regulation of gene transcription, cluster 3 included genes involved in reduction–oxidation reactions in the mitochondrion, cluster 4 included genes involved in methyltranferase activity, and cluster 5 included genes involved in cilium assembly. To identify enriched pathways, we performed the KEGG analysis. We confirmed 2317 genes were assigned by the KEGG analysis among genes with decreased DNA methylation in the tolerized T cells by 0.9-fold or less, using DAVID Bioinformatics Resources 6.8. The result showed that several pathways were changed in the tolerized T cells (Table 5). There are several pathways associated with diseases relevant to immune dysfunction, for example ‘Maturity onset diabetes of the young’ (Fold enrichment = 2.76, *p* = 2.07E-2) and ‘Autoimmune thyroid disease’ (Fold enrichment = 1.84, *p* = 3.69E-2). Next, we analyzed the genes with decreased methylation status in the tolerized T cells in more detail. We have shown the genes in Table 6. They were selected based on the following 4 criteria: 1) the fold change of the methylation rate of the gene decreased by 0.5-fold or less, 2) evaluated as significant by Fisher’s exact test, 3) the fold change of the count of methylated cytosine either decreased or remained unchanged, and 4) the sum of counts of methylated cytosine in the three samples of control T cell was 3 or more. This list includes several interesting genes, for example Rfx3 (FC = 4.99E-01, *p* = 1.89E-02), Tfcp2l1 (FC = 4.01E-01, *p* = 6.33E-03), Sp8 (FC = 1.02E-01, *p* = 2.44E-05), Ndnl2 (FC = 4.50E-01, *p* = 1.55E-02), and Ptgis (FC = 3.40E-01, *p* = 3.51E-02), which work for transcription regulation, and Sash3 (FC = 2.03E-01, *p* = 6.92E-03), Gimap1 (FC = 1.00E-01, *p* = 1.12E-02), and Mat1a (FC = 1.79E-01, *p* = 1.18E-02), which are involved in immune responses.

**Table 4:** Functional analysis of genes with decreased DNA methylation status during tolerization.

**Annotation Cluster 1**	**Enrichment Score: 1.2**		**Gene Counts**	*P* value*
	BP	smoothened signaling pathway	4	1.60E-02
	MF	sequence-specific DNA binding	11	1.90E-02
	MF	RNA polymerase II core promoter proximal region sequence-specific DNA binding	7	5.10E-02
	MF	transcription factor binding	6	1.10E-01
	BP	cell differentiation	10	1.30E-01
	MF	transcriptional activator activity, RNA polymerase II core promoter proximal region sequence-specific binding	5	1.40E-01
	BP	positive regulation of transcription from RNA polymerase II promoter	11	2.00E-01
	MF	transcription factor activity, sequence-specific DNA binding	10	2.10E-01
	BP	brain development	4	2.20E-01
	BP	multicellular organism development	11	2.30E-01
	BP	positive regulation of transcription, DNA-templated	5	6.20E-01
	CC	intracellular	6	9.90E-01
**Annotation Cluster 2**	**Enrichment Score: 1.01**		**Gene Counts**	***P* value**
	CC	transcription factor complex	7	1.50E-02
	MF	sequence-specific DNA binding	11	1.90E-02
	MF	DNA binding	20	8.60E-02
	BP	positive regulation of transcription from RNA polymerase II promoter	11	2.00E-01
	MF	transcription factor activity, sequence-specific DNA binding	10	2.10E-01
	BP	transcription, DNA-templated	17	3.20E-01
	BP	regulation of transcription, DNA-templated	19	4.20E-01
**Annotation Cluster 3**	**Enrichment Score: 0.77**		**Gene Counts**	***P* value**
	BP	oxidation-reduction process	10	6.40E-02
	MF	oxidoreductase activity	8	1.60E-01
	CC	mitochondrion	12	7.40E-01
**Annotation Cluster 4**	**Enrichment Score: 0.71**		**Gene Counts**	***P* value**
	MF	methyltransferase activity	4	1.30E-01
	BP	methylation	4	1.30E-01
	MF	transferase activity	11	6.60E-01
**Annotation Cluster 5**	**Enrichment Score: 0.7**		**Gene Counts**	***P* value**
	BP	cilium assembly	5	1.60E-02
	BP	cilium morphogenesis	5	3.80E-02
	CC	ciliary basal body	4	4.80E-02
	BP	cell projection organization	3	3.10E-01
	CC	cilium	3	5.70E-01
	CC	cell projection	5	7.80E-01
	CC	cytoskeleton	5	9.70E-01

The functions of genes with decreased DNA methylation status in the tolerized T cells were analyzed by DAVID Bioinformatics Resources 6.8. We found 167 such genes on the DAVID database and categorized them into 24 clusters. We have shown the top 5 among them in this table. MF: Molecular Function, BP: Biological Process, CC: Cellular Component.

*Statistical significance of gene enrichment was evaluated by *P* Value with Fisher’s exact test.

**Table 5:** The enriched KEGG pathways for decreased DNA methylation status in the tolerized T cells.

KEGG entry ID	KEGG pathway	Annotated gene number	*P* value*	Fold enrichment
mmu05202	Transcriptional misregulation in cancer	31	2.52E-03	1.75
mmu04740	Olfactory transduction	143	3.87E-03	1.23
mmu04950	Maturity onset diabetes of the young	8	2.07E-02	2.76
mmu00900	Terpenoid backbone biosynthesis	7	3.06E-02	2.83
mmu05320	Autoimmune thyroid disease	14	3.69E-02	1.84
mmu04976	Bile secretion	13	7.35E-02	1.70
mmu00980	Metabolism of xenobiotics by cytochrome P450	12	7.65E-02	1.75
mmu00140	Steroid hormone biosynthesis	15	7.87E-02	1.61
mmu00982	Drug metabolism—cytochrome P450	12	9.11E-02	1.69
mmu05323	Rheumatoid arthritis	14	9.63E-02	1.59

*Statistical significance of gene enrichment was evaluated by *P* Value with Fisher’s exact test.

**Table 6:** Specific genes with down-regulated DNA methylation status in the tolerized T cells.

Gene Name	Gene Name
enoyl-coenzyme A delta isomerase 3 (Eci3)	Gm11961
leucine zipper protein 1	Gm6851
transmembrane emp24 protein transport dmain containing 4	BC051142
prostaglandin I2 (prostacyclin) synthase (Ptgis)…A, B	Gm8369
insulin-like growth factor-binding protein 3	0610039K10Rik
NUS1 dehydrodolichyl diphosphate synthase subunit	Gm5591
THUMP domain containing 2	Gm44853
transcription factor CP2-like 1 (Tfcp2l1)…A	intersectin 2
congenital dyserythropoietic anemia, type I (human) (Cdan1)	Gm13603
SAM and SH3 domain containing 3 (Sash3)…B	Gm8595
ubiquitin specific peptidase 4 (proto-oncogene)	Gm4886
methionine adenosyltransferase I, alpha (Mat1a)…B	Gm13244
Ttk protein kinase	Gm12880
oocyte specific homeobox 4 pseudogene 18	Gm5538
fibrinogen-like protein 2	Gm20646
regulatory factor X, 3 (influences HLA class II expression) (Rfx3)…A	Gm14347
Tctex1 domain containing 4	defensin beta 5
trans-acting transcription factor 8 (Sp8)…A	Gm16531
hydrolethalus syndrome 1	Gm27003
vomeronasal 1 receptor pseudogene 104	Gm5710
coiled-coil domain containing 79	Gm28360
serine (or cysteine) peptidase inhibitor, clade B, member 9g (Serpinb9g)	Gm28115
necdin-like 2 (Ndnl2)…A	Gm37060
isthmin 1, angiogenesis inhibitor (Ism1)	Gm37401
olfactory receptor 1226	Gm43324
BBSome interacting protein 1	Gm43491
GTPase, IMAP family member 1 (Gimap1)…B	Gm8968
tau tubulin kinase 2	Gm44902
olfactory receptor 1329	

Each gene with decreased DNA methylation status in the tolerized T cells was selected based on several criteria.

Particular genes were marked as follows; A: genes related to control of gene expression, B: genes related to immune responses

### Several genes up-regulated in orally tolerized T cells had a decreased DNA methylation status

Finally, we showed 12 genes in Table 7 as candidates for the master regulator of orally tolerized T cells, selecting according to following 4 criteria: 1) the fold change of expression increased by 2.0 or more (S2 Table), 2) the fold change of expression was evaluated as significant by Fisher’s exact test (S2 Table), 3) the fold change of the methylation rate of the gene decreased by 0.9-fold or less (S4 Table), and 4) the fold change of the count of methylated cytosine either decreased or remained unchanged (S4 Table). The genes include Med9 (FC = 6.17E03, *p* = 1.59E-02 for expression; FC = 7.14E-01, *p* = 2.42E-01 for DNA methylation) involved in the regulation of transcription [16], Hist1h2ai (FC = 7.88E04, *p* = 4.75E-05 for expression; FC = 7.05E-01, *p* = 3.25E-01 for DNA methylation) involved in chromatin remodeling [17], and Lxn (FC = 3.85E03, *p* = 4.06E-02 for expression; FC = 7.05E-01, *p* = 4.13E-01 for DNA methylation) associated with inflammation [18]. On the other hand, most of them have not been reported yet if they have specific functions in anergic T cells.

**Table 7:** Candidate genes with epigenetically up-regulated expression in tolerized T cells.

Gene Name	Fold Change of expression (Anergy/Control)	Fold Change of methylation rate (Anergy/Control)	Fold Change of Number of methylation counts (Anergy/Control)
latexin (Lxn)	3852.90	0.775	0.714
Gm9725	4057.28	0.768	0.778
small nuclear ribonucleoprotein polypeptide F (Snrpf)	31159.55	0.856	0.786
histone cluster 1, H2ai (Hist1h2ai)	78820.71	0.705	0.800
synaptosomal-associated protein 29 (Snap29)	4276.21	0.882	0.800
coiled-coil domain containing 124 (Ccdc124)	32784.47	0.855	0.864
mediator complex subunit 9 (Med9)	6170.38	0.714	0.889
NHP2 ribonucleoprotein (Nhp2)	30765.92	0.832	0.960
histocompatibility 2, K region locus 2 (H2-K2)	78273.46	0.763	1.000
guanine nucleotide-binding protein (G protein), gamma 5 (Gng5)	51113.92	0.821	1.000
mitochondrial ribosomal protein S33 (Mrps33)	9583.20	0.835	1.000
Gm15429	12700.74	0.854	1.000

All of the genes with increased expression and down-regulated DNA methylation status were listed in this table.

### Most cytosine in CpG islands were methylated

We calculated the rate of methylated cytosine in CpG, CHG, and CHH sequences. Our data showed that most of methylated cytosines were found in CpG islands and the methylation rate of CpG islands was approximately 75% in both normal and tolerized T cells (Table 8).

**Table 8:** Location of the methylated cytosines in the control and tolerized T cells.

Treatment	Control			Tolerized		
Library ID	L563	L564	L565	L566	L567	L568
**Total number of methylated cytosine**	17,415,433	13,065,802	14,845,224	24,915,137	28,768,260	18,955,565
**Methylated cytosine in CpG (%)**	73.8	74.2	75.1	73.6	73.4	73.7
**Methylated cytosine in CHG (%)**	0.9	0.7	0.9	0.8	0.7	0.6
**Methylated cytosine in CHH (%)**	1.1	0.9	1.0	0.9	0.9	0.8

The location of the methylated cytosines in the DNA prepared from the control and tolerized T cells was analyzed by Bismark software. H: adenine, cytosine, or thymine

## Discussion

T cell anergy is known to be an important phenomenon that is involved in several types of immune tolerance, such as self-tolerance, superantigen-induced tolerance, and oral tolerance [1–3,19]. Therefore, many studies have been conducted to clarify the molecular mechanisms for unresponsiveness of anergic T cells [20,21] albeit with little success. In the present study, we aimed to identify the genes which are specifically expressed *via* epigenetic regulation in anergic T cells obtained from orally tolerized mice. Given that the expression of so many genes in T cells gets altered during tolerization, it is believed that one or few key molecule(s) get specifically expressed in anergic T cells, similarly with T-bet, GATA-3, RORγt, and Foxp3 for Th1, Th2, Th17, and Treg cells [9–11], respectively, and then regulate the expression of other genes. Some of these master regulators have been demonstrated to be controlled under epigenetic modification of DNA [12]. A comprehensive analysis of anergy-specific gene regulations would contribute to the fine understanding of molecular mechanisms of T cell tolerization.

By transcriptome analysis, we detected 2034 up-regulated genes and 2286 down-regulated genes in orally tolerized T cells. This result shows that up-regulated genes were fewer than down-regulated genes, supporting the fact that various types of responses are suppressed in tolerized T cells. A similar result was observed in our previous study in which we had used T cells anergized *in vitro* [13].

The result of DAVID analysis indicated that clusters 1 and 2 included genes involved in chromatin remodeling. For example, PAPA-1 (listed in Table 3) is a subunit of INO80, which is known to induce chromatin remodeling by DNA binding [22]. This result suggests that the epigenetic regulation of the gene expression may be important to maintain the hyporesponsiveness of the cells. Interestingly, clusters 3 and 4 included various genes involved in positive immune responses, such as cytokine and chemokine activities, suggesting that some T cells included in the tolerized T cell population are not completely unresponsive and can influence other cells around them in some specified way. This result is supported by the study of Zheng *et al*. showing that some anergic T cells could enhance peripheral tolerance *via* interactions with other immune cells [23]. In addition, some pathways were indicated by KEGG analysis to be altered in tolerized T cells, including ones important for immune regulation (Table 2). Therefore, dysregulation of such critical pathways would result in deficiency of T cell function. Particularly, TGF-β is known to be one of the important cytokine involved in oral tolerance. The change in TGF-β-related signaling pathway would play a key role in the tolerized T cells. All things considered, the results of DAVID and KEGG analysis suggests that the data obtained from the transcriptome analysis are reliable because the genes found to be up-regulated are valid as anergy-related genes.

The genes listed in Table 3 included several interesting genes, such as CD52, Hspe1, Ire3, and C1qbp. These genes have been reported as negative regulators of immune responses. For example, the proliferation and IFN-γ production of T cells that highly express CD52 were shown to be inhibited [24]. The differentiation of T cells into Th17 cells and the production of IL-17 were reportedly enhanced in Ire3-deficient mice [25]. These evidences suggest that those genes contribute to maintaining the hyporesponsiveness of tolerized T cells.

Table 3 also shows that several genes involved in transcription regulation were up-regulated in the tolerized T cells. Some of them (Rest and Lbh) directly regulate gene transcription as transcription factors, while others indirectly control it *via* binding to transcription factors (Yaf2, Chchd2, C1qbp, and Hexim1) or regulating the activity of RNA polymerase II (Med10, Med9, and Cks2). These genes may include the key molecules that regulate the expression of various genes specific to anergy induction in T cells.

The next-generation sequencing technology made it possible to comprehensively understand the DNA methylation status of animal cells. We detected about 20,000,000 methylcytosine residues, and most of them were found in CpG islands (Table 8). The methylation rate of CpG islands was approximately 75%. This result shows that the data are reliable because it has been generally known to be 60%–90% in mammalian cells.

Our result showed that the genes with decreased methylation rate caused by oral tolerance were fewer than those with increased methylation rate (Fig 3), thus supporting the overall result demonstrating that genes with increased expression were fewer than those with decreased expression (Fig 2). As described above, these changes may be partly involved in hyporesponsiveness of tolerized T cells. For example, in a previous study, expression of IL-2 was reported to be regulated by the methylation status of specific CpG sites located in an area within approximately 600 bp of the promoter region [26].

Conversely, our result also showed that the methylation rate of 285 genes was down-regulated during tolerization, suggesting that the expression of some of these genes was up-regulated by epigenetic regulation. It is suggested that several specific methylation sites would particularly contribute to control the expression of genes. For example, the differentiation into Treg cell was critically determined by the demethylation of TSDR on the Foxp3 gene [12]. It is considered that the methylation status of CpG sites on the binding regions of RNA polymerases or transcription factors was particularly important for regulating gene expression. Therefore, in future studies, we need to analyze the change in methylation rate focusing on each cytosine to better understand the regulatory mechanism of the altered gene expression, using by other more efficient strategies, such as microarray-based DNA methylation analysis [27] and Agilent SureSelect Mouse Methyl-Seq [28]. We do not have sufficient capacity to perform that using the data of the genome wide DNA methylation analysis with bisulfite sequencing at present because such analysis needs a computer with higher performance and deeper coverage of data. On the other hand, the results obtained from this study according to the average change of methylation status within 10 kb upstream from the TSS are sufficiently reliable to achieve the aim of this study. It has been demonstrated that CpG islands at these regions have critical roles for regulating gene expression.

The result of DAVID analysis for demethylated genes indicated that clusters 1 and 2 included the genes involved in transcription regulation, such as Rfx3, Sp8, and Tfcp2l1, which are listed in Table 6. There is no previous report on the immunoregulatory functions of these genes, although Rfx reportedly works in pancreatic β cells [29] and Sp8 plays a role in the development of neural cells under epigenetic regulation [30]. On the other hand, Table 6 also includes several genes involved in immune responses. Sash3 regulates cytokine production [31], Gimap1 controls B and T cell differentiation [32], and Ptgis is associated with the suppression of NFκB activity [33]. Conversely, the functions remain unknown for approximately a half of genes listed in Table 6. The identification of their functions would greatly contribute understanding the molecular mechanism of T cell anergy.

The KEGG analysis indicated that DNA methylation status of genes involved in several pathways was altered in the tolerized T cells (Table 5). Several of them suggested dysfunction of immune regulation, for example ‘Maturity onset diabetes of the young’ and ‘Autoimmune thyroid disease’. These diseases are induced by abrogation of immune tolerance. Therefore, the alteration of the pathways in the orally tolerized T cells suggests the importance of those pathways in oral tolerance as well. However, how the change contributes to unresponsiveness of the tolerized T cells is needed to clarify in future investigation. Interestingly, the results of KEGG analysis shown in Tables 2 and 5 included a common pathway ‘Transcriptional misregulation in cancer’. Supplementary figure (S1 Fig) illustrated the pathway. The genes mapped in the pathway were indicated as red stars. This pathway gives the cancer cells specific characteristics such as differentiation resistance and survival. These features are also likely to be seen in tolerized T cells.

**Fig 4:**
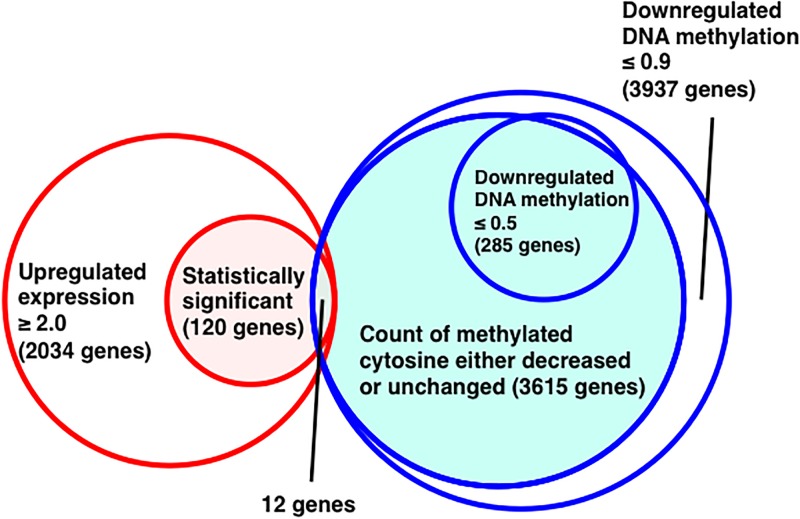
Procedure to pick up the genes with up-regulated expression and down-regulated DNA methylation. Genes with significantly increased expression (≥ 2.0) and decreased methylation rate (≤ 0.9) in tolerized T cells. In addition, the count of methylated cytosine of the genes either decreased or unchanged in the cells.

We adopted several other criteria to pick up genes which were the candidates for specifically expressed genes *via* epigenetic regulation in orally tolerized T cells (Table 7) because we could not detect the expression of most genes listed in Table 6 by the transcriptome analysis. Briefly, we expanded target genes with the fold change of methylation rate decreased by 0.9-fold or less because only few specific methylation sites might be important to control the expression of the gene. We summarized the procedure to pick up the genes shown in Table 5 (Fig 4). These genes are expected to encode key molecules for maintaining the anergic state. Med9 was the only gene known as a transcription factor among the genes listed in Table 7. Med9 is a subunit of a mediator complex which interacts with the carboxyl domain of the major subunit of RNA polymerase II and regulates the expression of specific genes by acting as a transcription co-activator [16]. This evidence suggests the involvement of Med9 in the regulation of genes important for anergy induction. The experiments for revealing the function of Med9 in anergic T cells are ongoing. In addition, an experiment for obtaining more data regarding the expression of the genes listed in Table 6 is also now in progress. It is expected that we would find other candidate genes for key molecules through the experiment. Then, the experiment to analyze the function of the newly identified candidates as well as the genes in Table 7 will be performed.

One of the aims of this study was to compare the result from T cells tolerized *in vivo* with that from T cells anergized *in vitro*. As shown in Table 9, we found only few genes of which expression were up-regulated in both *in vitro* anergized and *in vivo* tolerized T cells. The number of the genes is less than expected, suggesting that the mechanism for inducing anergy would not always be the same for anergic T cells induced *in vitro* and *in vivo*. On the other hand, it cannot be denied that these few genes play critical roles in the induction of T cell anergy. In addition, the methylation status of Snrpf, Ccdc124, Med9, Gng5, and Gm15429 among the genes listed in Table 7 was also comparably decreased in *in vitro* anergized T cells (Snrpf; FC = 0.655, Ccdc124; FC = 0.627, Med9; FC = 0.733, Gng5; FC = 0.333, Gm15429; FC = 0.464), suggesting that the change of methylation status of those genes would be regulated by a common mechanism in both cells, and such mechanism is crucial for inducing T cell anergy. Further research is needed to identify the common mechanism and the molecules involved in it.

**Table 9:** Genes up-regulated in both *in vitro* anergized and *in vivo* tolerized T cells.

Gene Name	Fold Change of expression *in vitro* (Anergy/Control)	Fold Change of expression *in vivo* (Anergy/Control)
RIKEN cDNA 1700021F05 gene	1.54	4147.15
coiled-coil domain containing 64B	1.59	118.74
Gm16284	1.51	115.34
granzyme D	1.51	61.49
ermin ERM-like protein	1.52	11.47
Gm10800	1.82	4.025
acyl-CoA thioesterase 13	1.66	3.29
sin3 associated polypeptide	1.55	2.95
prostaglandin reductase 1	1.62	2.22
solute carrier family 1 (glutamate/neutral amino acid transporter) member 4	1.53	2.17
SLAM family member 9	1.56	2.13
Gm13936	1.65	2.05

Data for *in vitro* anergized T cells were quoted from our previous study [13].

Several anergy-specific genes, such as GRAIL, Itch, Cbl-b, Egr2, and Egr3, reportedly play important roles in anergy induction [4–7, 34, 35]. Indeed, we confirmed the expression of GRAIL was certainly up-regulated by inducing oral tolerance (Fig 1D). In this study, we could analyze the methylation status of these genes, except for that of GRAIL; however, the methylation status of these genes did not change even after tolerization (Itch; FC = 1.441, Cbl-b; FC = 1.169, Egr2; FC = 1.261, Egr3; FC = 1.168), indicating that the expression of these genes is probably regulated by mechanisms other than DNA demethylation. Similar results were obtained in our previous study using T cells anergized *in vitro* as well. It is suggested that these genes are not the master regulators of anergic T cells, and other key molecules with epigenetically enhanced expression may control those genes.

We were wondering if the transcriptome and the comprehensive DNA methylation analysis could be applied for T cells tolerized *in vivo* because each T cell was tolerized in various degree *in vivo* compared to T cell anergized *in vitro*. In addition, T cells orally tolerized *in vivo* would include not only anergic T cells but also other distinct populations such as Treg cells. Contamination of those cells might have interfered the analysis even if they are not major population. Furthermore, purity of antigen-specific T cells used in this study was also a big concern. Contamination of antigen-nonspecific T cells and non-T cells might also have interfered the analysis. However, in this study, we could identify several genes specifically expressed and epigenetically regulated in orally tolerized T cells, suggesting that the methods used in this study was enough to find candidates for the master regulator while can be improved to obtain more precise data. Most of those genes identified in this study have not been previously reported as candidates for the master regulator of anergic T cells. These results could be obtained thanks to the comprehensive analysis using next-generation DNA sequencing technology. This strategy can be applied for the investigation of master regulators of other unique cells. This study would contribute not only to clarifying the mechanism of T cell anergy but also for understanding various biological events.

## Supporting information

S1 Table The number of reads obtained from DNA sequencing for transcriptome analysis. Each cDNA library was prepared from the DNA samples obtained from the control and tolerized T cells. They were analyzed with Illumina HiSeq sequencer.
(XLSX)

S2 Table Genes of which expression were upregulated in orally tolerized T cells. (XLSX)

S3 Table The number of reads obtained from DNA sequencing for DNA methylation analysis. Each DNA library was prepared from the DNA samples obtained from the control and tolerized T cells after bisulfite treatment. They were analyzed with Illumina GAIIx sequencer.
(XLSX)

S4 Table Genes of which DNA methylation were down-regulated. (XLSX)

S1 Fig The KEGG pathway commonly altered among genes with enhanced expression and decreased DNA methylation. (A) Enhanced expression, (B) decreased DNA methylation.
(PPTX)

S2 Fig Graphical abstract. (TIFF)
