# Supplementary figures and images for: Comprehensive analysis of DNA methylation and gene expression in orally tolerized T cells
Source: PLoS One. 2020 Feb 25;15(2):e0229042. doi: 10.1371/journal.pone.0229042 (PMC7041840; doi:10.1371/journal.pone.0229042)

## Slide 1
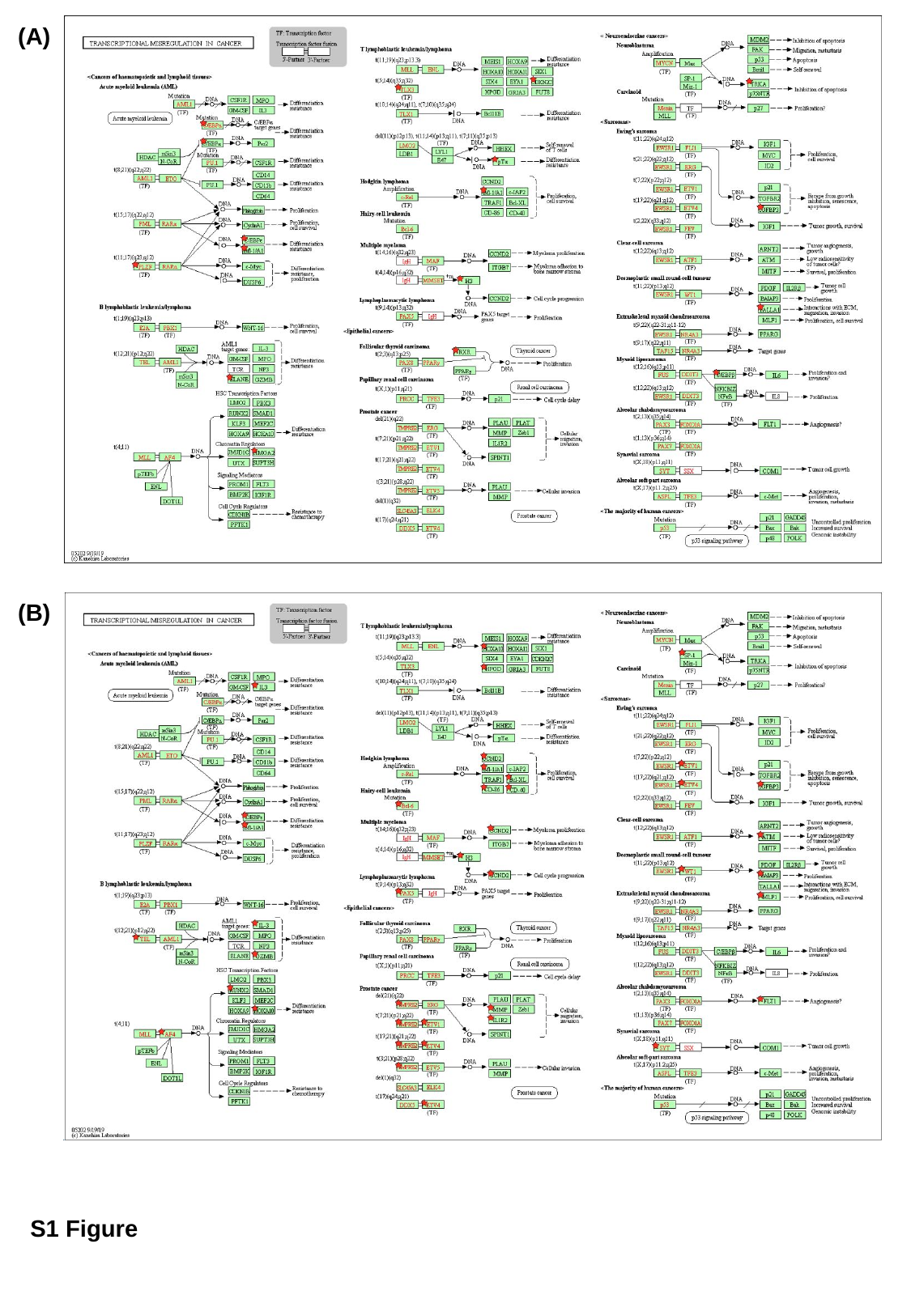

(A)
(B)
S1 Figure

Supplement: S1 Fig — (A) Enhanced expression, (B) decreased DNA methylation. (PPTX) [file pone.0229042.s005.pptx]

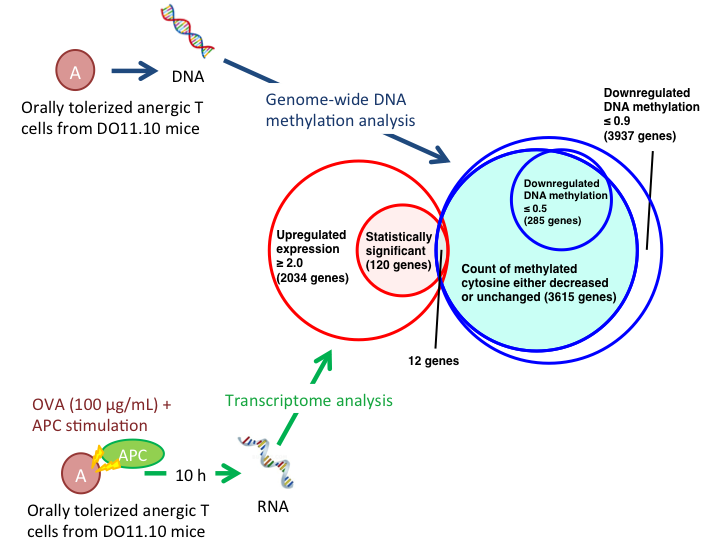

Supplement: S2 Fig — (TIFF) [file pone.0229042.s006.tiff]
